# Regulation of PKC Autophosphorylation by Calponin in Contractile Vascular Smooth Muscle Tissue

**DOI:** 10.1155/2013/358643

**Published:** 2013-11-19

**Authors:** Hak Rim Kim, Cynthia Gallant, Kathleen G. Morgan

**Affiliations:** ^1^Department of Health Sciences, Boston University, 635 Commonwealth Avenue, Boston, MA 02215, USA; ^2^Department of Pharmacology, College of Medicine, Dankook University, 119 Dandaero, Chungnam, Cheonan-si 330-714, Republic of Korea

## Abstract

Protein kinase C (PKC) is a key enzyme involved in agonist-induced smooth muscle contraction. In some cases, regulatory phosphorylation of PKC is required for full activation of the enzyme. However, this issue has largely been ignored with respect to PKC-dependent regulation of contractile vascular smooth muscle (VSM) contractility. The first event in PKC regulation is a transphosphorylation by PDK at a conserved threonine in the activation loop of PKC, followed by the subsequent autophosphorylation at the turn motif and hydrophobic motif sites. In the present study, we determined whether phosphorylation of PKC is a regulated process in VSM and also investigated a potential role of calponin in the regulation of PKC. We found that calponin increases the level of in vitro PKC*α* phosphorylation at the PDK and hydrophobic sites, but not the turn motif site. In vascular tissues, phosphorylation of the PKC hydrophobic site, but not turn motif site, as well as phosphorylation of PDK at S241 increased in response to phenylephrine. Calponin knockdown inhibits autophosphorylation of cellular PKC in response to phenylephrine, confirming results with recombinant PKC. Thus these results show that autophosphorylation of PKC is regulated in dVSM and calponin is necessary for autophosphorylation of PKC in VSM.

## 1. Introduction

In recent years, it has become clear that multiple, redundant signaling pathways are responsible for the fine-tuning of smooth muscle contractility [1]. This complexity allows close control of important physiologic processes such as blood pressure and blood flow; however, it also raises the question as to how the cell regulates the spatially precise activity of overlapping pathways [2, 3]. Especially of interest is the fact that protein kinase C (PKC), a family of serine/threonine kinases, has the potential to activate many cellular substrates in vitro and yet only discrete pathways are activated in the cell. 

PKC isoforms are grouped into 3 classes based on the domain composition of the N-terminal half of the molecule [4–6]. The C1 domain is a diacylglycerol (DAG) sensor and the site of binding of phosphatidyl serine (PS). The C2 domain contains the calcium sensor. Conventional PKCs (cPKC) are activated by Ca^2+^, DAG, and PS. Novel PKCs (nPKC) have a nonligand binding C2 domain and are activated by DAG and PS but not Ca^2+^. Atypical PKCs contain a nonligand binding C1 domain and are activated by PS but by neither Ca^2+^ nor DAG. The conventional PKC*α* and the novel PKC*ε* are the best studied isoforms in the contractile, fully differentiated vascular smooth muscle cell (dVSMC) [7–10]. In the last decade, accumulating evidence from in vitro protein chemistry and cellular studies of nonmuscle cell types has indicated that regulatory phosphorylation of PKC itself is required for full activation of PKC [4], but, this issue has largely been ignored with respect to PKC regulation in contractile dVSM.

 In general, all members of the AGC kinase family are now thought to require a series of ordered phosphorylations that are thought to “prime” the kinase and convert it into a mature form capable of being activated by the allosteric activators. The first event is a phosphorylation by PDK at a conserved threonine in the activation loop [11, 12]. This is thought to introduce a negative charge that properly aligns residues to form a competent catalytic domain and to facilitate the subsequent autophosphorylation at 2 sites in the C-terminus at the “turn motif”, so named because it corresponds to a phosphorylation site in PKA localized at the apex of a turn, and the more C-terminal “hydrophobic motif” which comprises a Ser flanked by bulky hydrophobic residues [13–15]. These events are thought to keep the enzyme in a catalytically competent and protease resistant conformation. Full activation of PKC by allosteric activators is thought to induce an open conformation that makes the enzyme susceptible to both proteases and phosphatases, thus either leading to repeated autophosphorylation/dephosphorylation cycles [14] or to proteolytic degradation and the requirement for new synthesis of the enzyme [4, 14].

The phosphorylation of PKC is thought by some to occur during maturation of the newly synthesized enzyme [16–18]. However, some controversy has ensued regarding whether phosphorylation of PKC is dynamically regulated. Studies of PKC*α* in a variety of cell types in culture have been consistent with the completion of such phosphorylation events only during enzyme maturation; however, for novel PKCs at least 2 groups have reported that the phosphorylations are dynamically regulated [12, 16, 19]. A study using NIH 3T3 cells reported that PKC phosphorylation increases in response to PDGF [16]. Also, PKC*ε* and PKC*δ* in cardiomyocytes in primary culture appear to undergo regulated and regulatory phosphorylation of the activation loop and the hydrophobic motif even in the absence of allosteric regulators [12]. Thus the regulatory pathways may be isoform-specific as well as cell-type specific.

Protein kinase C is a key enzyme involved in signaling during smooth muscle contraction [20]. Previously, our group has found that an abundant smooth muscle differentiation marker, calponin (CaP), can activate PKC in vitro in the absence of lipid cofactors [21]. Furthermore, acute knockdown of CaP protein levels inhibits PKC-dependent contractility in vascular smooth muscle [22], and CaP has been shown to coimmunoprecipitate with PKC after *α*-agonist induced activation vascular tissue [23]. This raises the question of whether CaP can also promote activation of PKC in vivo in vascular tissue. Thus, the present studies were performed to determine if phosphorylation of PKC is a regulated process in dVSM and to investigate a potential role for CaP in the regulation of PKC activation.

## 2. Materials and Methods

### 2.1. In Vitro Protein Kinase Assay

PKC phosphorylation was carried out in the following solution: A: 150 mM NaCl, 10 mM imidazole, 2 mM MgCl_2,_ 0.01% NaN_3_, 1 mM ATP, and 15 mM *β*-mercaptoethanol. Recombinant PKC*α* or recombinant PKC*ε* (Panvera) was incubated in solution with either 25 *μ*M BSA, 25 *μ*M protamine, or 25 *μ*M calponin for 15 seconds at 37°C. Calponin was prepared from turkey gizzard. The reaction was stopped by the addition of sample buffer followed by heating. The phosphorylation of PKC was detected by western blot using specific site phosphorylation antibodies.

### 2.2. Tissue Preparation and Force Measurements

All procedures in this study were performed according to protocols approved by the Institutional Care and Use Committee. Ferrets (Marshall Farms, North Rose, NY) were euthanized with either chloroform or isoflurane in a ventilation hood. The aorta was quickly removed and immersed in oxygenated 95% O_2_-5% CO_2_ physiological salt solution (PSS) composed of (in mM) 120 NaCl, 5.9 KCl, 25 NaHCO_3_, 11.5 dextrose, 1 CaCl_2_, 1.4 MgCl_2_, and 1.2 NaH_2_PO_4_, pH 7.4. The aorta was cut into circular strips, attached to a force transducer, and allowed to equilibrate for at least 1 hour before being challenged with a depolarizing saline solution replacing 51 mM NaCl with 51 mM KCl, to confirm viability and for normalization. The tissues were washed and allowed to relax for 1 hour before beginning the experiment. The muscle strips were frozen at the desired time points following agonist stimulation by using a dry ice acetone, TCA slurry, and stored at −80°C for further study. Calponin knockdown was performed by 4 days of exposure of vascular strips to phosphorothioate antisense or a scrambled version of the ferret CaP sequence (5′-AT GTT TTC CAG CTG GTG CCA A-3′), or a scrambled version of that sequence (5′-CG TGG TAT AAA ACC GAT CAC G-3′) in serum free organ culture, as previously describe [22].

### 2.3. Immunoblotting

The previously frozen samples were homogenized at 4°C in a buffer containing 50 mM Tris-HCl, 10% glycerol, 140 mM NaCl, and 1% Triton X-100. Sample homogenates were subjected to immunostaining. Blots were visualized with a supersignal west peroxide solution (Pierce). The images were detected with a chemiluminescence imaging screen with a Bio-Rad molecular Imager phosphor imager and quantified with Multi-Analyst software or with an Odyssey infrared imaging system (LI-COR Biosciences).

### 2.4. Antibodies

A phosphospecific antibody against the activation loop of PKCzeta (Thr^410^) and which cross-reacts with the activation-loop Thr^497^ of all PKCs (Upstate Biotechnology) detected phosphorylation of PKC at the PDK site (1 : 500). A phosphospecific antibody against PKC Thr^638^ of PKC (Cell Signaling) detected the phosphorylation of PKC at turn motif (1 : 2000). A phosphospecific antibody against Ser^719^ of PKC (Upstate biotechnology) detected the phosphorylation of PKC*ε* at hydrophobic site (1 : 1000). A phosphospecific antibody against Ser^657^ of PKC (Upstate biotechnology) detected the phosphorylation of PKC at hydrophobic site (1 : 1000). Alexa Fluor 680 goat anti-mouse IgG and Alexa Flour 680 goat anti-rabbit IgG were all from Molecular Probes. IR-Dye 800 goat anti-rabbit IgG and IR-Dye 800 goat anti-mouse IgG were both from Rockland.

### 2.5. Statistical Analysis

All values given in the text are expressed as mean ± SE. The *n* values represent the number of animals used in experiments. Data were compared by using a Student's *t*-test with probability values of *P* < 0.05 considered significant.

## 3. Results

### 3.1. Calponin Increases the Level of PKC*α* Phosphorylation at the PDK and Hydrophobic Sites, but Not the Turn Motif Site In Vitro

**Figure 1:**
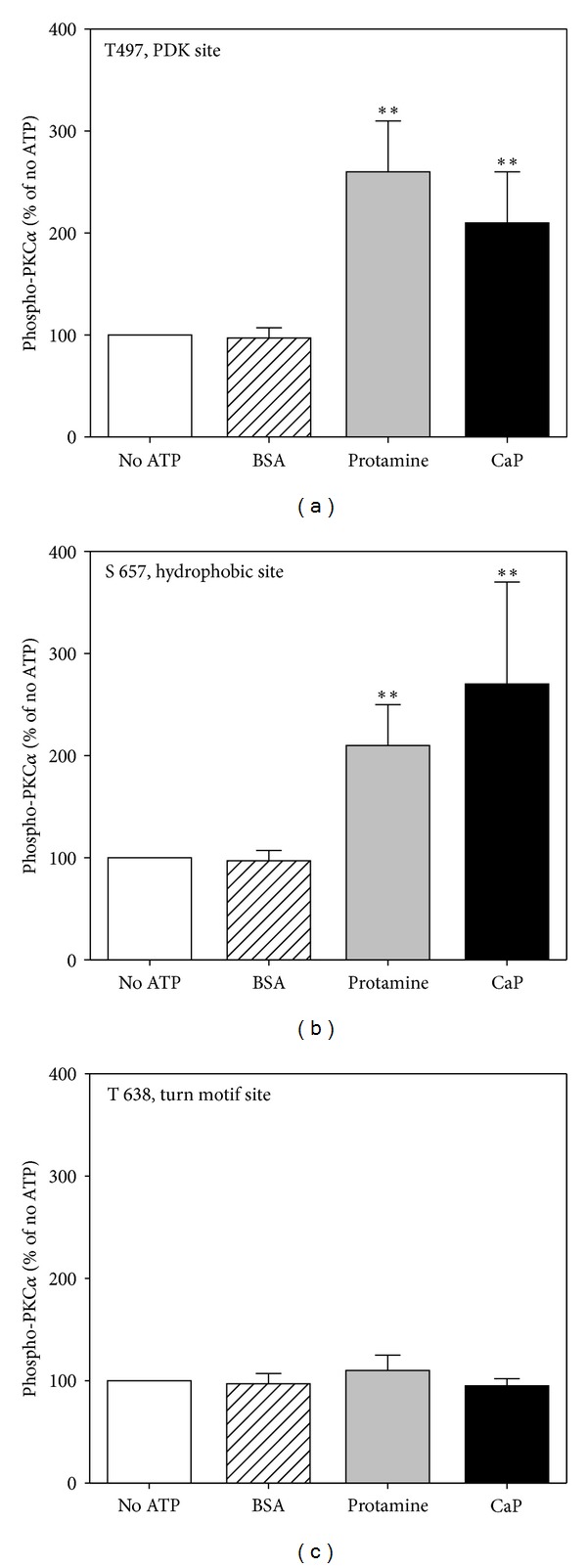
Calponin increases autophosphorylation on the PDK and hydrophobic sites but not the turn motif site of recombinant PKC. (a) Mean densitometric results of 6 experiments using a site-specific phosphoantibody to the PDK site (T497) as described in methods. (b) Mean densitometric results of 6 experiments, phosphorylation detected with a site-specific phosphor-antibody to the hydrophobic site (S657). (c) Mean densitometric results of 6 experiments, phosphorylation detected with a site-specific antibody for the turn motif site (T638). No ATP and BSA lanes represent negative controls. Protamine is a positive control. Data are mean ± SE. *P < 0.05 and **P < 0.01 versus No ATP.

We have previously reported [21] and confirmed here that the addition of calponin to PKC invitro in the presence of ATP, but the absence of lipids, increases the amount of phosphorylation at the PKC*α* hydrophobic site (Figure 1(b)). We now report that the addition of calponin also increases phosphorylation at T497, the putative PDK transphosphorylation site, (detected with a site specific phosphoantibody) 2-3-fold over that in the absence of ATP (Figure 1(a)). In contrast, the addition of an equimolar amount of BSA as a negative control had no effect. The addition of an equimolar amount of protamine produced similar levels of activation to that caused by calponin. Protamine is used as a positive control since it is also known to be able to activate PKC in the absence of lipid cofactors [24, 25]. In contrast, as can be seen in Figure 1(c), the addition of calponin in vitro does not significantly change the level of phosphorylation of PKC*α* at the turn motif site (T638). Interestingly, protamine, a known direct activator of PKC [24, 25], also had no effect on the phosphorylation at this site.

### 3.2. PKC Hydrophobic Site, but Not Turn Motif Site, Phosphorylation Increases in an Agonist-Dependent and Time-Dependent Manner in Contractile dVSM

**Figure 2:**
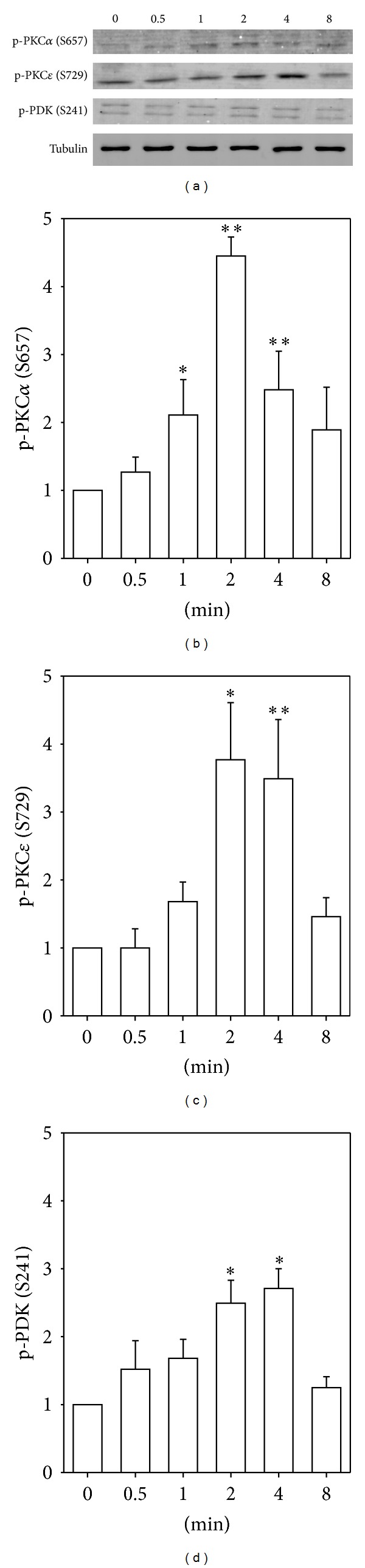
(a) Representative immunoblots of PKC*α* and PKC*ε* hydrophobic site phosphorylation, and PDK phosphorylation, in PE-stimulated ferret aorta samples, by using phosphospecific antibodies. (b)–(d) Quantitation of changes of phosphorylation levels of PKC*α*, PKC*ε*, and PDK after stimulating with PE (10 *μ*M) in ferret aorta. (b) Time course of phosphorylation at hydrophobic site of PKC*α* (S657). (c) Time course of phosphorylation at hydrophobic site of PKC*ε* (S729). (d) Time course of phosphorylation of PDK (S241). Data are mean ± SE; *n* = 3–7 animals. *P < 0.05 and **P < 0.01 compared with control (0 minutes).

Aortic tissue strips were quick-frozen at different time points after the addition of the vasoconstrictor phenylephrine (10^−5^ M), and the level of PKC phosphorylation at the hydrophobic and turn motif sites was determined by immunoblot (Figure 2). Both PKC*ε* and PKC*α* have been shown to play a role in the regulation of contractility of smooth muscle tissue [7–10] and to be present in aorta, with ferret aorta containing abundant levels of PKC*ε* and lesser levels of PKC*α* [7, 8, 26].  Figure 2(a) shows typical blots in 2 minutes PE-stimulated samples. Complete time courses are quantitated in Figures 2(b) and 2(c), and it can be seen that addition of the *α*-agonist, phenylephrine, causes a time dependent increase, up to 3-fold, in the immunodetection of phosphorylation of aortic PKC*α* and PKC*ε* at the hydrophobic site. Thus, phosphorylation of the PKC hydrophobic site in dVSM is clearly regulated, rather than constitutive after maturation of the enzyme. A band was also seen with a turn motif phosphor-antibody at the correct molecular weight for PKC*α*, but no change in the level of phosphorylation of the turn motif site was detectable (data not shown).

### 3.3. PDK Is Activated in Response to *α*-Agonist Stimulation of Smooth Muscle Tissue

The antibody used to detect phosphorylation of recombinant PKC at the PDK site in vitro was raised against PKC zeta sequence, but sufficiently cross-reacted with purified PKC*α* that it could be used for in vitro studies. However, when used against a whole cell aorta homogenate, multiple cross-reacting bands obscured the results. Thus, in order to estimate the time course of PDK activation, we monitored phosphorylation of PDK itself at S241 in the activation loop. The anti-phospho-PDK antibody detected immunoreactive proteins with appropriate molecular masses of 58 and 68 kDa [27, 28]. As is shown in Figure 2(d), phenylephrine addition significantly increases the level of phosphorylation of PDK at this site by 2 minutes. 

### 3.4. Activation of *α*-Adducin at S662, Caldesmon at S789, ERK 1/2 in Response to *α*-Agonist Stimulation in Smooth Muscle Tissue

**Figure 3:**
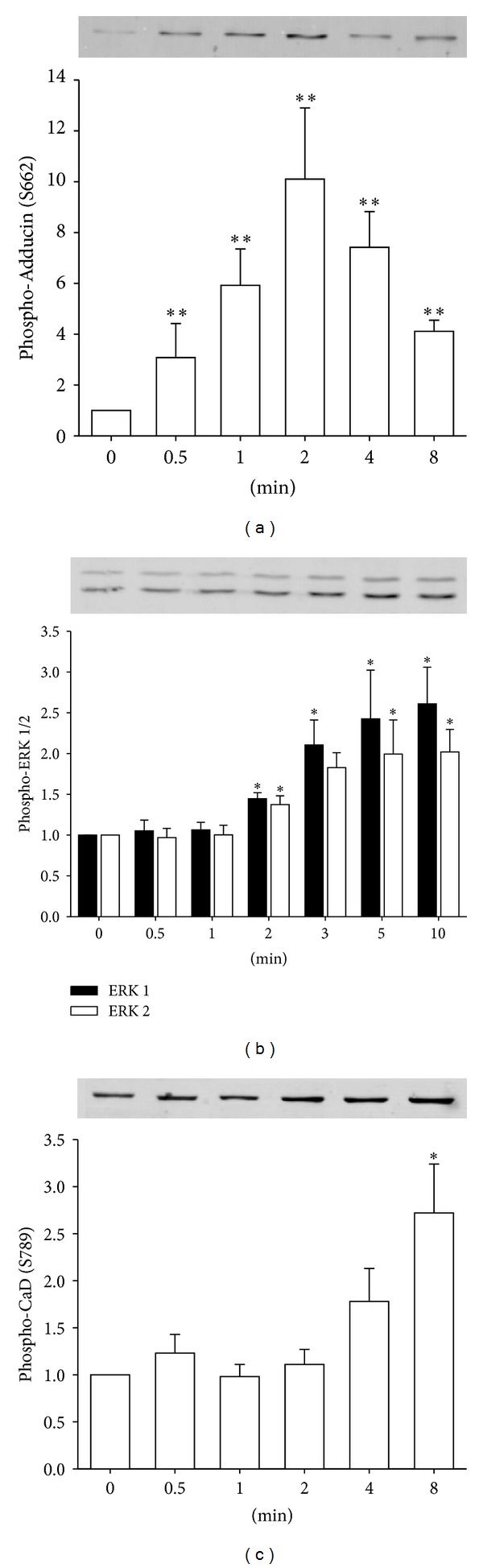
Temporal lag between phosphorylation of known direct PKC substrate and downstream PKC-dependent signaling events in aorta samples. (a) Time course of phosphorylation of known intracellular PKC substrate, *α*-Adducin after activating tissue with PE (10 *μ*M). Data are mean ± SE; *n* = 4–7 animals. **P < 0.01 compared with control (0 minute). (b) ERK 1/2 after addition of PE (10 *μ*M). (c) Time course of phosphorylation of caldesmon. Data are mean ± SE; *n* = 4–7 animals. *P < 0.05 and **P < 0.01 compared with control (0 minutes).

To determine the cellular time course of the onset of actual kinase activity of PKC in vivo, we monitored the phosphorylation level of 3 proteins in dVSM. First, adducin is a ubiquitously expressed calmodulin-binding protein and substrate for protein kinase C. S662 on *α*-adducin is known to be a specific PKC substrate in most tissues and has been used as an assay of in-cell PKC activation [29, 30]. We used it here since the direct PKC substrate in these cells is not known. As is shown in Figure 3(a), the phosphorylation of *α*-adducin is detectable at 30 seconds and peaks at 2 minutes after the addition of phenylephrine (10^−5^ M) to ferret aorta. Secondly, ERK 1/2 is known to be downstream of PKC in a pathway activated by phenylephrine in this tissue [21, 31]. Phosphorylation of ERK 1/2 is detectable at 2 minutes but peaks at 5–10 minutes after addition of PE (Figure 3(b)). Thirdly, caldesmon is known to be phosphorylated directly by ERK 1/2 at S789 in the same PE-activated pathway in this dVSM tissue. The phosphorylation of CaD is detectable at 8 minutes after stimulating aorta with PE (Figure 3(c)). Thus the phosphorylation of both PKC*α* and PKC*ε*, at the hydrophobic site, peaking at 2 minutes after PE addition has an appropriate timecourse to cause the activation of PKC sufficiently early to lead to the subsequent direct phosphorylation of *α*-adducin as well as the downstream signaling to ERK and CaD (Figure 3).

### 3.5. Calponin Knockdown Inhibits the Autophosphorylation of PKC in dVSM

**Figure 4:**
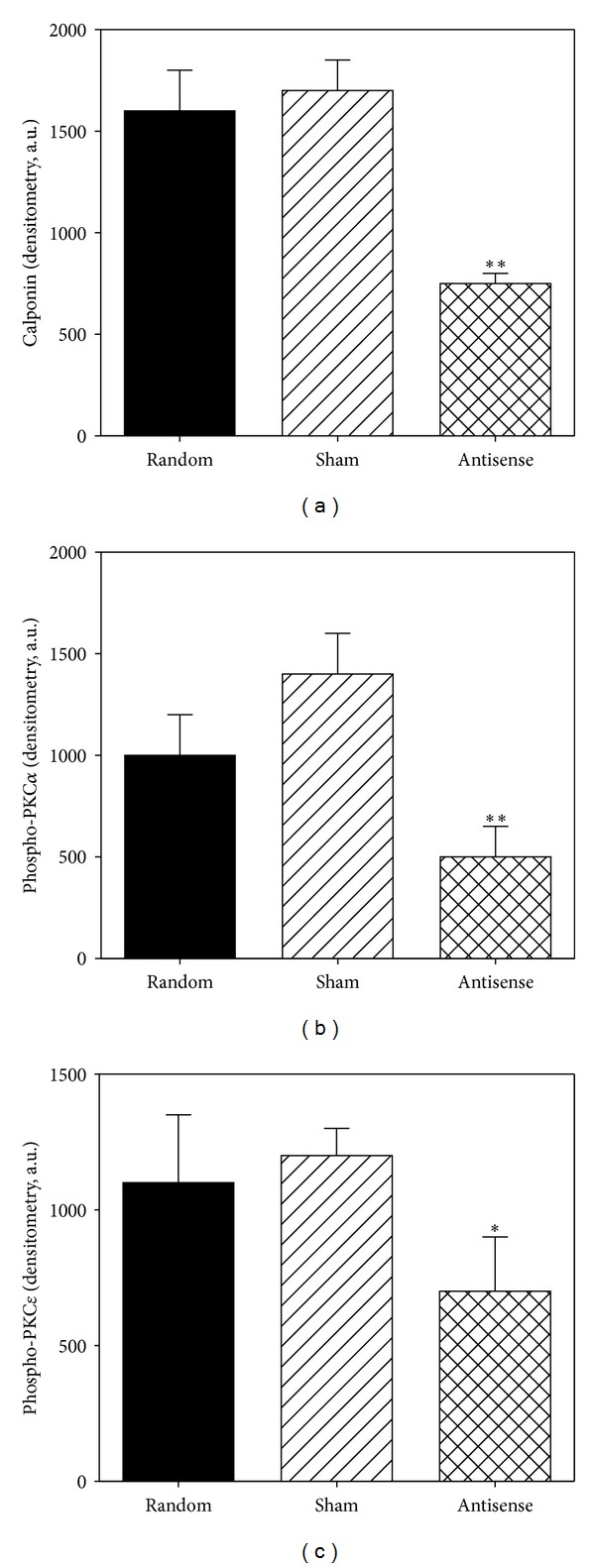
CaP knockdown in aorta dVSM inhibits phosphorylation of PKC*α*, PKC*ε*. (a) Densitometric analysis of calponin protein levels after knockdown. (b) Decreased phosphorylation of PKC*α* in the presence of PE (10 *μ*M) with CaP knockdown. (c) Decreased phosphorylation of PKC*ε* in the presence of PE (10 *μ*M) with CaP knockdown. Data are mean ± SE; *n* = 4–7 animals. *P < 0.05 and **P < 0.01 compared with sham control.

Since CaP facilitates the phosphorylation of PKC at the hydrophobic site in vitro and since CaP and PKC have been shown to coimmunoprecipitate in an agonist-dependent manner [23] and to cotranslocate to the cell cortex during *α*-agonist induced activation [23] as well as the findings above that the timecourse of PE-induced hydrophobic site phosphorylation of PKC is sufficiently rapid to cause activation of the PKC-ERK-CaD pathway in this tissue, we tested the idea that CaP-dependent facilitation of PKC activation is a necessary step in this pathway in dVSM by knocking down CaP protein levels with an antisense approach in serum-free organ culture of aortic tissue strips (Figure 4). 

As can be seen in Figure 4(a), CaP protein levels were decreased in antisense loaded strips (by ~50% compared to sham or random loaded samples, resp.). PKC*α* hydrophobic site phosphorylation was measured in phenylephrine-treated quick frozen muscles and was found to be significantly decreased in the calponin-knockdown strips (decreased 63% of that in sham loaded strips and 66% of that in random sequence loaded strips) (Figure 4(b)). In parallel, the PKC*ε* hydrophobic site phosphorylation levels were also measured and found to be significantly decreased in the calponin-knockdown strips (Figure 4(c)). Thus, taken together with previous results demonstrating a coimmunoprecipitation of CaP with PKC [23], these results demonstrate that an interaction of CaP with PKC is necessary for *α*-agonist-induced phosphorylation and activation of PKC in dVSM.

### 3.6. Activation with the *α*-Agonist, Phenylephrine, Sequentially Increases Phosphorylation of ERK1/2, *α*-Adducin, and CaD in dVSM

**Figure 5:**
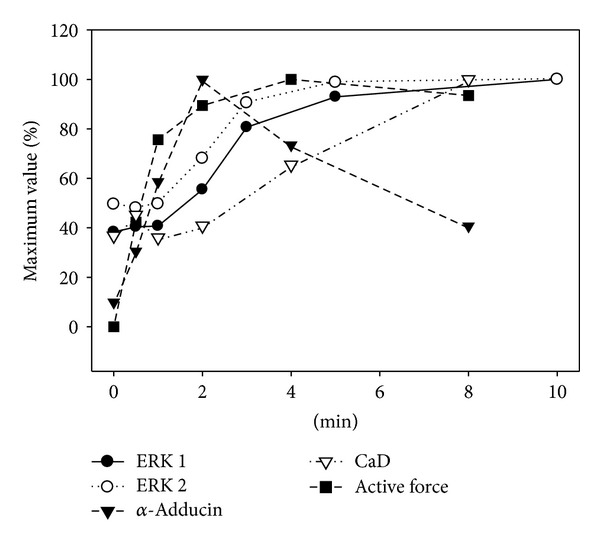
Time course of PE-induced changes in contractility and phosphorylation of ERK1/2, *α*-adducin, and caldesmon. In each case, the mean results were normalized as a percentage of the average maximal increase above baseline (*n* = 4–7). The *x*-axis is in minutes after the addition of PE (10 *μ*M). SE bars have been deleted for clarity.

 It is of interest to compare the relative temporal profile of signaling events reported here and in past work on this same tissue, aorta of the ferret. We have previously reported that there is a largely transient increase in intracellular ionized Ca^2+^ levels and subsequent myosin light chain phosphorylation levels peaking at 30 sec ~ 1 min after the addition of PE in this tissue [32, 33] which is expected to cause the early rise in contractile force at the one-minute-time point (Figure 5). Although force begins to rise slightly before other events measured in the present study, it is known that steady state force amplitude is dependent on PKC activity in many smooth muscle tissues [34, 35]. The first of the signaling events to reach a peak level shown here is *α*-adducin (Ser662) which has been monitored previously as a surrogate reporter of cellular PKC kinase activity [29, 30, 36]. As is shown in Figures 2 and 3, phosphorylation of both PKC*α* and PKC*ε* at the hydrophobic site occurs, within the resolution of our methods, at essentially the same time point, consistent with PKC autophosphorylation causing full activation of the kinase.

Of interest also is the fact that subsequent events previously shown to be downstream of PKC activation in this tissue, ERK1/2 phosphorylation and CaD phosphorylation (Ser789) reach the maximum much later, with CaD phosphorylation being the slowest event. Thus, additional factors are clearly involved, perhaps including the fact that PKC is activated at the cell membrane whereas smooth muscle CaD is spatially removed, residing on the contractile filaments in the core of the cell. The phosphorylation of *α*-adducin does not show this delay, presumably because it is associated with the membrane cytoskeleton [37].

## 4. Discussion

The main findings of the present study are that autophosphorylation of PKC*α* and PKC*ε* is regulated by an *α*-agonist in dVSM and that the smooth muscle differentiation marker, CaP, is necessary for this autophosphorylation. We have previously shown that CaP is necessary for maximal *α*-agonist-induced contraction of aorta [22] and that *α*-agonists trigger a translocation of CaP from the contractile filaments to the cortex of the dVSMC [38]. CaP coimmunoprecipitates from aorta tissue with PKC as well as ERK1/2 and directly binds to both PKC and ERK1/2 in vitro [21, 23, 31]. These past findings resulted in the hypothesis that CaP functions as a scaffolding protein for PKC and ERK [23], but it has not been clear if CaP plays a role in the activation of PKC in vivo. Here we have demonstrated that regulation of PKC occurs in dVSM when activated by an *α*-agonist and that CaP is necessary for this process.

Ca^2+^/CaM/myosin light chain kinase (MLCK) mediated phosphorylation of the 20 kDa myosin light chains (LC20) is an important mechanism of regulation of smooth muscle contraction and is known to be tightly controlled by regulation of both MLCK and myosin phosphatase by multiple mechanisms [1]. It has previously been shown that a parallel, PKC activated pathway in aorta also regulates contractility. This pathway leads to activation of ERK1/2 and phosphorylation of its downstream substrate, CaD. The CaD is an actin binding protein that, in conjunction with tropomyosin, interferes with the availability of actin for interaction with myosin [39, 40]. Phosphorylation of CaD reverses this inhibitory action [41]. Apparently this PKC-dependent pathway is also tightly regulated in space and time by the previously demonstrated translocations of CaP, ERK, and PKC and the necessary role of CaP, shown here, for activation of the kinase activity of PKC via autophosphorylation.

There are three key phosphorylation sites for PKC functions, known as the activation loop, hydrophobic-motif, and turn-motif. Interestingly, PDK was phosphorylated and PKC hydrophobic motif phosphorylation was increased by phenylephrine stimulation in dVSM, but there were no detectable changes in the level of phosphorylation of the turn motif site in this study, neither invitro nor in vivo. Many studies reported that phosphorylation of the activation loop, catalyzed by the PDK, is critical for activation of PKC. Mutation of phosphorylatable residues in the activation loop abolished PKC activity [42, 43]. However, the turn motif which is located in the C-terminal tail is dispensable for catalytic activity of some isoforms of PKC [44, 45]. 

To further determine the time course of the onset of actual kinase activity of PKC in vivo, we monitored the phosphorylation level of *α*-adducin, reported to be a PKC-specific substrate that can be used as an in-cell monitor of PKC kinase activity [29, 30, 46]. When used here in dVSMCs, it is of interest that the phosphorylation of *α*-adducin occurs quite early, but the phosphorylation of CaD is delayed by several minutes and presumably is due to time delays involved in the yet to be determined process by which ERK moves from the cell cortex, where it is activated, to the location of the ERK substrate, CaD, on the contractile filaments in the core of the cell. The delay of several minutes is surprising from a kinetic point of view but may point to diffusion slowed by the viscosity of the cytosol in contractile smooth muscle, multiple ERK binding sites in the cell and other ERK sequestering mechanisms yet to be determined.

Thus, in summary, the present study demonstrates that the vasoconstrictor, phenylephrine activates, in aortic dVSM, a pathway involving CaP-dependent PKC autophosphorylation and activation followed by a much-delayed ERK activation, CaD phosphorylation and contraction. This PKC-dependent pathway occurs in parallel with the previously described transient spike in intracellular Ca^2+^ levels and subsequent myosin light chain phosphorylation in this tissue [23, 47, 48], demonstrating the true complexity of signaling at the whole cell level.
